# An analog of photon-assisted tunneling in a periodically modulated waveguide array

**DOI:** 10.1038/srep35744

**Published:** 2016-10-21

**Authors:** Liping Li, Xiaobing Luo, Xiaoxue Yang, Mei Wang, Xinyou Lü, Ying Wu

**Affiliations:** 1 School of Physics, HUST, Wuhan 430074, People’s Republic of China; 2 Department of Physics, Jinggangshan University, Ji’an 343009, People’s Republic of China

## Abstract

We theoretically report an analog of photon-assisted tunneling (PAT) originated from dark Floquet state in a periodically driven lattice array without a static biased potential by studying a three-channel waveguide system in a non-high-frequency regime. This analog of PAT can be achieved by only periodically modulating the top waveguide and adjusting the distance between the bottom and its adjacent waveguide. It is numerically shown that the PAT resonances also exist in the five-channel waveguide system and probably exist in the waveguide arrays with other odd numbers of waveguides, but they will become weak as the number of waveguides increases. With origin different from traditional PAT, this type of PAT found in our work is closely linked to the existence of the zero-energy (dark) Floquet states. It is readily observable under currently accessible experimental conditions and may be useful for controlling light propagation in waveguide arrays.

Controlling quantum tunneling and transport through a periodic driving field has been a subject of intense studies in the last decades, for its relevance to fundamental physics tests as well as to great potential application in nanoscale devices^1^,^2^. Among the most intriguing aspects of the subject, coherent destruction of tunneling (CDT)^3^ and photon-assisted tunneling (PAT)^4^ represent two seminal results. CDT is a resonant effect discovered in the pioneering work, in which the coherent tunneling between states is almost completely suppressed when the system parameters are carefully chosen at the isolated degeneracy point of quasi-energies^3^. It has so far generated great interests and has recently been observed experimentally in different physical systems^5^,^6^. Recently, CDT has been found to occur over a wide range of system parameters in odd-*N*-state systems where one state is periodically driven with respect to others^7^. Such extension of destruction of tunneling to a finite parameter range, referred to as dark CDT, is attributed to the existence of localized dark Floquet state with zero quasi-energy^7^,^8^,^9^. Introduction of dark Floquet state and dark CDT, which are hitherto limited in the high-frequency regime, may offer benefits for all-optical switching and coherent quantum control.

Photon-assisted tunneling (PAT) refers to a phenomenon in which tunneling contact disabled by a static tilt (dc bias potential) can be restored when the system exchanges energy of an integer number of photons with the oscillating field^10^. The static tilt (dc bias potential) leads to suppression of tunneling which is related to localized Wannier-Stark states^11^. When a multiple of the driving frequency of ac field matches the energy difference between adjacent rungs of the Wannier-Stark ladder, the system is able to absorb or emit photons with sufficient energy to bridge the energy difference created by the dc bias potential, through which tunneling is (partly) restored (PAT). The appealing concept of PAT originated in the prototype system with a quantum particle confined in a driven Wannier-Stark lattice. Recently it has found growing theoretical interest in many-body dynamics of bosonic systems^12^,^13^,^14^,^15^,^16^. So far, PAT has been experimentally observed in Josephson junctions^17^, coupled quantum dots^18^,^19^, semiconductor superlattices^20^,^21^ and Bose-Einstein condensates in optical lattices^22^.

In this article, we have studied the tunneling dynamics in lattice arrays with controllable boundary. Owing to the simplicity and flexibility offered by optical settings, the engineered photonic waveguides provide an ideal system for exploration of tunneling phenomena, in which spatial propagation of light mimics the temporal dynamics of a quantum particle in a lattice array^23^,^24^. Generally, PAT occurs in a system with a static biased potential which strongly suppresses usual Josephson oscillations. But here we report an analog of photon-assisted tunneling in a periodically driven lattice array without static tilt (dc bias potential) by comprehensively studying a three-channel waveguide system. Our numerical analysis discovers that dark CDT (strong suppression of tunneling) and dark Floquet state still exist in the three-channel waveguide system even in the non-high-frequency regimes where the modulation frequency of the periodically modulated top waveguide is roughly equal to or smaller than the coupling strength between the bottom and its adjacent (middle) waveguide. However, when integer multiples of the modulation frequency approximately equal to the coupling strength between the bottom and its adjacent (middle) waveguide, the light tunneling from the top waveguide to the others is restored as a clear signature of photon-assisted tunneling. In our previous two works (refs 7 and 8), we have addressed a novel extension of coherent destruction of tunneling (CDT) and its application for coherent control. In the current work, however, we report a series of photon-assisted tunneling (PAT) resonances in the considered model by moving the last waveguide (site) closer to its neighbor. Different from the PAT observed in the earlier studies which usually requires a static biased potential to initialize the system in a self-trapped state, this type of PAT is closely linked to a dark Floquet state with zero quasi-energy. Our results are applicable for the five-channel waveguide system and also extendable to waveguide arrays with an odd number of waveguides.

## Results

### The physical model for periodically modulated waveguide system

**Figure 1:**
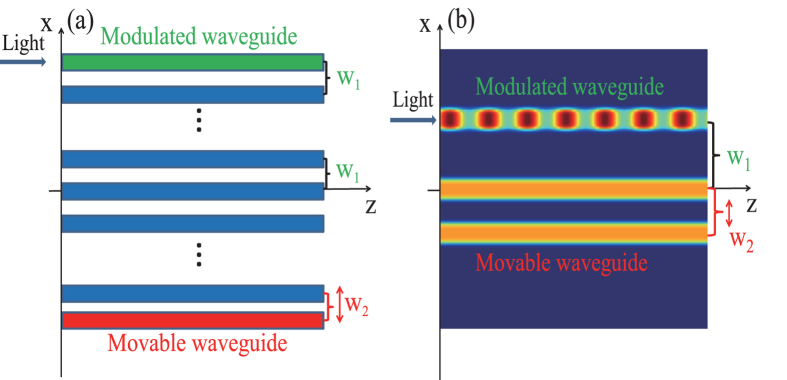
Schematic diagram of the modulated waveguide system. (**a**) Schematic of a tunneling-coupled optical waveguide array with controllable boundary that realizes an analog of photon-assisted tunneling. (**b**) A typical triplet waveguide system. The refractive index of the top boundary waveguide is modulated periodically along the propagation direction. The space separation between the bottom boundary waveguide and its nearest neighbor *w*_2_ is adjustable by moving the bottom boundary waveguide towards the other waveguides, while the spacings *w*_1_ between other nearest-neighboring waveguides are fixed.

As illustrated in Fig. 1, we present an optical implementation of our Hamiltonian in the form of a linear array of tunneling-coupled optical waveguides which is characterized in: (i) that the refractive index of the top boundary waveguide is modulated periodically along the propagation direction; and (ii) that the distance *w*_2_ between the bottom boundary waveguide and its nearest neighbor is different from other identical nearest-neighboring spacings *w*_1_. Thus, through adjustment of the distance *w*_2_, the coupling strength between the bottom boundary waveguide and its nearest neighbor can be tuned to be sufficiently large in comparison to the modulation frequency. The role of photon is played by a periodic modulation of the the top boundary waveguide with a certain modulation frequency. With the use of the coupled-mode approach, the optical-field dynamics in such structures are described by the following set of equations^9^:


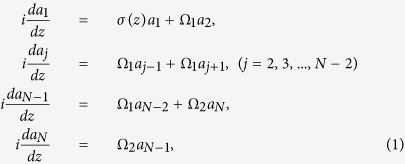


where *a*_*j*_ is field mode amplitude in the *j*-th waveguide, *z* the propagation distance, Ω_1_ the coupling strength between neighboring waveguides with spacing *w*_1_, Ω_2_ the coupling strength between the bottom boundary waveguide (*j* = *N*) and its adjacent waveguide (*j* = *N*−1), and *σ*(*z*) the normalized difference between the propagation constants of the top boundary waveguide and the other waveguides of the array. As in ref. 9, instead of modulating an array of waveguide in a uniform fashion, modulating one certain waveguide selectively is implemented here. We consider a harmonic modulation of the linear refractive index of the top boundary waveguide along the propagation direction with *σ*(*z*) = *A* sin (*ωz*), where *A* is the relative depth of the harmonic longitudinal modulation, and *ω* is the spatial modulation frequency. Such a periodic modulation is well within the capacity of current experiments^23^,^24^. In a different perspective, the above Equation (1) can be regarded as describing the system of a quantum wave in a periodically driven lattice array if *z* is viewed as time *t*. As is well known, the periodic time-dependent equation (1) admits solutions in the form of Floquet states 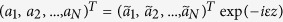, where *ε* is the quasi-energy and the amplitudes 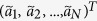 are periodic with modulation period *T* = 2*π*/*ω*.

### PAT in three-guide system

We start our consideration for the three-guide system, the minimal one for odd-*N*-state systems. In this case, the dynamical equations are of the form


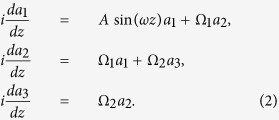


**Figure 2:**
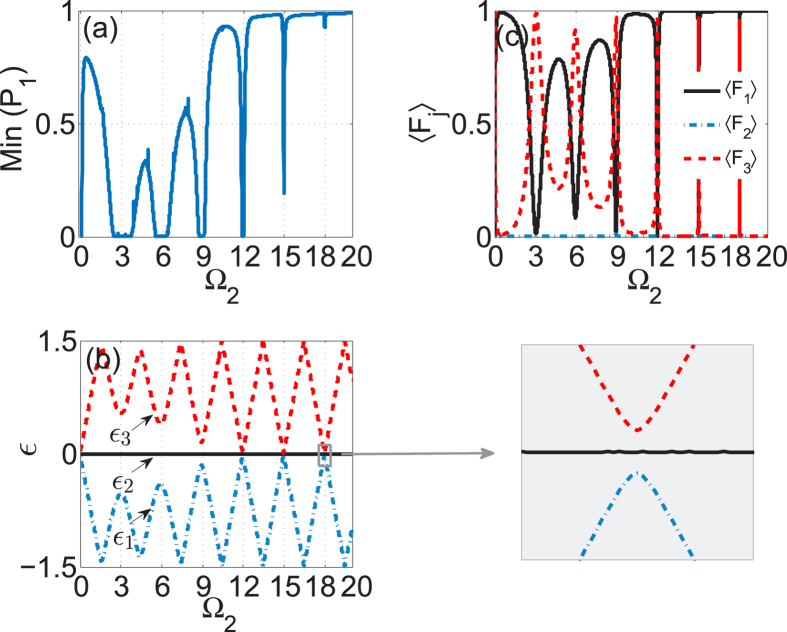
PAT in three-guide optical system. (**a**) the minimum value of intensity of light at the initially populated guide-1, Min(*P*_1_) versus Ω_2_, with *A* = 6.6, *ω* = 3, Ω_1_ = 1; (**b**) the corresponding quasi-energy *ε* versus Ω_2_ with no level degeneracy and (**c**) the time-averaged population 〈*F*_*j*_〉 belonging to the zero-energy (dark) Floquet state versus Ω_2_.

To study the system’s beam dynamics, we solve numerically the coupled-mode equations with the light initially localized in the 1-th waveguide (the top boundary waveguide). With the numerical solution, we compute the intensity of light staying in the initial waveguide by *P*_1_(*z*) = |*a*_1_(*z*)|^2^ and measure the minimum value of *P*_1_(*z*) over a long-enough propagation distance. When Min(*P*_1_) is not zero, the tunneling is suppressed as the light is not allowed to be fully transferred from the 1-th mode (guide) to the other modes (guides). In Fig. 2(a), we display Min(*P*_1_) versus the coupling strength Ω_2_ at the fixed parameters *A* = 6.6, *ω* = 3, Ω_1_ = 1. For Ω_2_ = 0, the system is in fact a two-guide system in which the conventional CDT happens only at the isolated degeneracy point of the quasi-energies, and consequently Min(*P*_1_) takes a zero value because the ratio of driving amplitude and frequency is set with small deviation from the isolated degeneracy point. When Ω_2_ is increased from zero, the value of Min(*P*_1_) becomes relatively large except at a series of very sharp dips. In general, periodic modulation of the top boundary waveguide will yield a significant suppression of the light tunneling in the three-guide system even with Ω_1_ ≠ Ω_2_, as shown in Fig. 2(a). However, at particular values of the coupling strength Ω_2_, Ω_2_ ≈ *nω* with *n* being integer, the value of Min(*P*_1_) exhibits a series of sharp dips, in analogy to the *n*-photon-like resonances which destroy the effect of suppression of tunneling. It also can be observed that as the coupling strength Ω_2_ is increased, the higher photon-like resonances become very weak and thus are almost not visible.

For a deep insight into the tunneling dynamics obtained in Fig. 2(a), we numerically compute the quasi-energies and Floquet states of this system as shown in Fig. 2(b,c). As shown in Fig. 2(b), this three-state system always possesses a Floquet state with zero quasi-energy regardless of the value of Ω_2_. The other two quasi-energies make a set of close approaches to each other as Ω_2_ is increased. At the points of close approach, namely, at Ω_2_ ≈ *nω*, the value of Min(*P*_1_) displays sharp dips and the tunneling is significantly restored. We also plot the time-averaged population distribution 
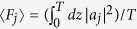
 for the zero-energy Floquet state (*a*_1_, *a*_2_, *a*_3_)^*T*^ in Fig. 2(c). Considering that the dynamic is determined by the Floquet states, self-trapping (suppression of tunneling) of light intensity initially populating at the 1-th mode (guide) will take place if 〈*F*_1_〉 > 0.5 holds. As seen in Fig. 2(c), the zero-energy Floquet state has negligible population at the central mode (guide) while the population 〈*F*_1_〉 is much larger than 0.5 for all values of Ω_2_ except those in the vicinity of Ω_2_ ≈ *nω*. Correspondingly, suppression of tunneling (CDT) occurs for all values of Ω_2_ except the locations of photon resonances, as shown in Fig. 2(a). The Floquet state with zero quasi-energy is essentially the dark Floquet state, not only for its zero quasi-energy but also for its negligible population at the central waveguide; the suppression of tunneling (CDT) is of the dark CDT as it is caused by the dark Floquet state rather than level degeneracy (as seen in the inset on the right side of Fig. 2(b)). In fact, the CDT-PAT transition found in Fig. 2(a) is closely related to the sharp localization-delocalization transition of population 〈*F*_1_〉 for the zero-energy (dark) Floquet state. Note that the dark Floquet state originally discovered and defined in the high-frequency limit can be reduced to the well-known dark state by means of high-frequency averaging method^7^,^8^. However, the dark Floquet state and the associated CDT can still exist in the non-high-frequency regimes where the coupling strength Ω_2_ is much larger than the modulation frequency and the high-frequency averaging method is invalid. These results will greatly enrich our understanding of dark Floquet state and dark CDT.

**Figure 3:**
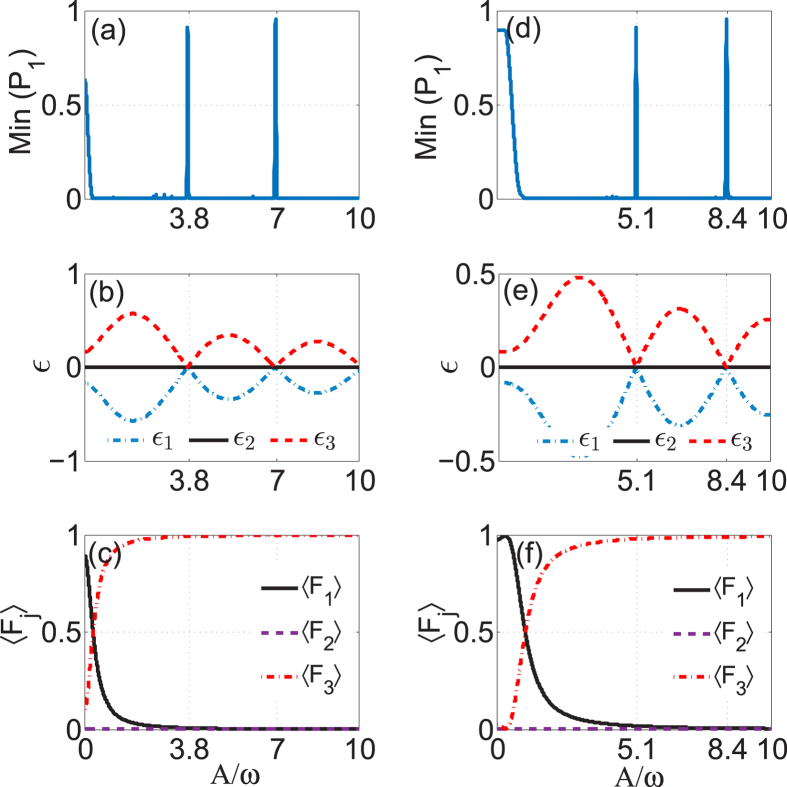
Transition between PAT and CDT for one-photon and two-photon resonances in three-guide optical system. The left column: (**a**) Min(*P*_1_) versus *A*/*ω* for the 1-photon resonance *ω* = 3, Ω_2_ = 3, Ω_1_ = 1; (**b**) the corresponding quasi-energy *ε* versus *A*/*ω* and (**c**) the time-averaged population 〈*F*_*j*_〉 belonging to the zero-energy (dark) Floquet state versus *A*/*ω*. The right column: (**d**) Min(*P*_1_) versus *A*/*ω* for the 2-photon resonance *ω* = 3, Ω_2_ = 6, Ω_1_ = 1; (**e**) the corresponding quasi-energy *ε* versus *A*/*ω* and (**f**) the time-averaged population 〈*F*_*j*_〉 belonging to the zero-energy (dark) Floquet state versus *A*/*ω*.

To get further study of *n*-photon-like resonances, we show how the value of Min(*P*_1_) varies under conditions that the modulation amplitude is increased, while its frequency is held constant at *ω* = 3 and the coupling strength held Ω_2_ = *nω*, Ω_1_ = 1. At multiphoton resonances, the tunneling is restored in general. However, CDT will occur at certain values of the amplitude of the driving field^10^,^12^. It means possibility of moving the system between PAT and CDT through variation of the amplitude of the driving field. Figure 3(a) shows the case of *n* = 1 photon resonance. For *A* = 0, the system is self-trapped in the 1-th waveguide due to the existence of an imbalanced dark state (−Ω_2_/Ω_1_, 0, 1)^*T*^ with Ω_2_/Ω_1_ > 1, and thus the value of Min(*P*_1_) is nonzero. When the periodic driving is applied, as *A* is increased from zero, the value of Min(*P*_1_) rapidly drops to zero, which indicates that the photon resonance destroys the self-trapping effect. When A/*ω* is increased further, Min(*P*_1_) takes extremely low values about zero except at a sequence of very narrow peaks. These peaks are precisely centered at *A*/*ω* = 3.83, 7.01, .…, the zeros of *J*_1_(*A*/*ω*). In Fig. 3(b,c), we plot the quasi-energies and the population distributions of the dark Floquet state for the first photon resonance (*n* = 1). Apparently, the quasi-energies are degenerate when *J*_1_(*A*/*ω*) = 0, and when away from *A* = 0 the dark Floquet state has averaged population at the 1-th mode (guide) well below the value of 0.5. Therefore it can be concluded that the well-defined quasienergy crossings instead of the dark Floquet state are the origin of the extremely sharp peaks (CDT resonances) seen in Fig. 3(a). The occurrence of CDT resonances centered at the degeneracy points of quasienergies (zeros of Bessel functions) is a feature commonly found in the periodically driven systems^10^,^12^, even in the case that the conditions of multiphoton resonances are satisfied.

In Fig. 3(d), we show the values of Min(*P*_1_) in the three-guide optical system for the *n* = 2 resonances. As is expected, the values of Min(*P*_1_) exhibit a number of extremely sharp peaks centered on the zeros of *J*_2_(*A*/*ω*) where the quasi-energies will be degenerate; see Fig. 3(e). Similar to the case of *n* = 1 resonance, the sharp peaks in the *n* = 2 resonance is also caused by the level degeneracy rather than the dark Floquet state, as it is shown in Fig. 3(f) that the population 〈*F*_1_〉 belonging to the dark Floquet state is well below 0.5 at the points of quasi-energy crossings.

**Figure 4:**
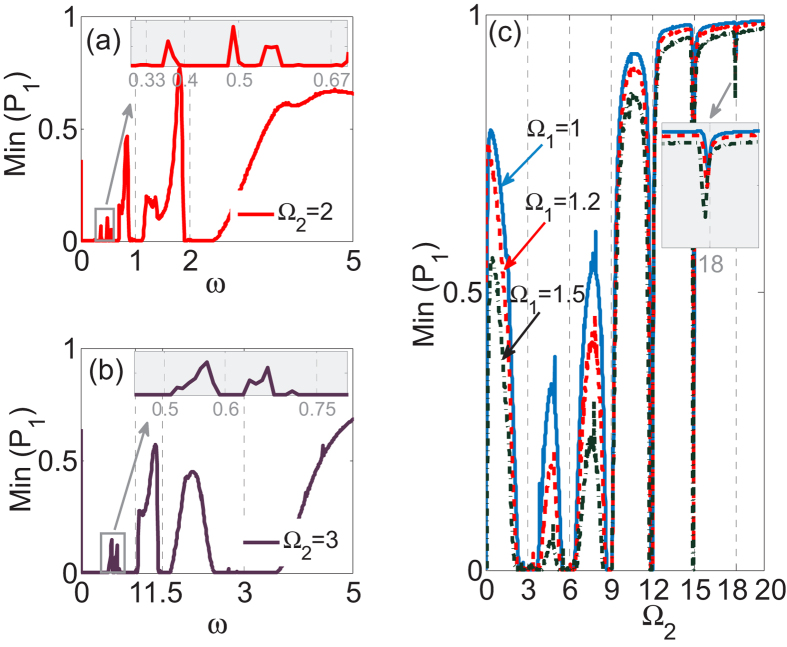
Dependence of PAT location on modulation frequency *ω* and coupling strength Ω_1_. (**a**,**b**) The minimum value of population distribution *P*_1_, Min(*P*_1_), as a function of the modulation frequency *ω* with Ω_2_ = 2 and Ω_2_ = 3 respectively. Other parameters are chosen as Ω_1_ = 1 and *A* = 6.6. (**c**) Min(*P*_1_) versus Ω_2_ with different values of Ω_1_. Other parameters are chosen as *ω* = 3 and *A* = 6.6.

In order to observe the *n*-photon-like resonances from a different angle, we also plot Min(*P*_1_) as a function of the modulation frequency *ω* for two fixed parameters Ω_2_ = 2 and Ω_2_ = 3 in Fig. 4(a,b) respectively, for the case of the system parameters Ω_1_ = 1 and *A* = 6.6. We can readily observe that the *n*-photon-like resonances occur at comparatively broad interval around *ω* = Ω_2_/*n*. The width of such photon-assisted tunneling resonances is much larger than those of PAT resonances observed in the literature. We now elaborate the physics underlying this photon-assisted tunneling resonances. The actual resonance condition does not refer directly to Ω_2_ = *nω* but rather to the tunneling frequency of the model (2) without periodic modulation. The unmodulated three-guide optical system admits three energy level as 
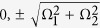
, and the space of two neighboring energy levels is 
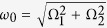
. In such a system, the existence of imbalanced dark state with zero energy results in the suppression of tunneling when the periodic modulation is switched off. The periodic modulation effectively creates “photons” that bridge the energy gap between neighboring energy levels. Thus, a photon-assisted tunneling resonance can occur at a modulation frequency which satisfies the resonance condition *ω*_0_ = *nω*. When Ω_2_ is considerably larger than Ω_1_, the energy difference *ω*_0_ of the unmodulated system will become principally characterized by Ω_2_ and therefore the resonance condition is approximately given by Ω_2_ = *nω*. As clearly seen in the inset in Fig. 4(c), the position of *n*-photon-like resonance does slightly shift with increasing Ω_1_ due to the dependence of the energy difference (tunneling frequency) *ω*_0_ on Ω_1_.

### PAT in five-guide system

**Figure 5:**
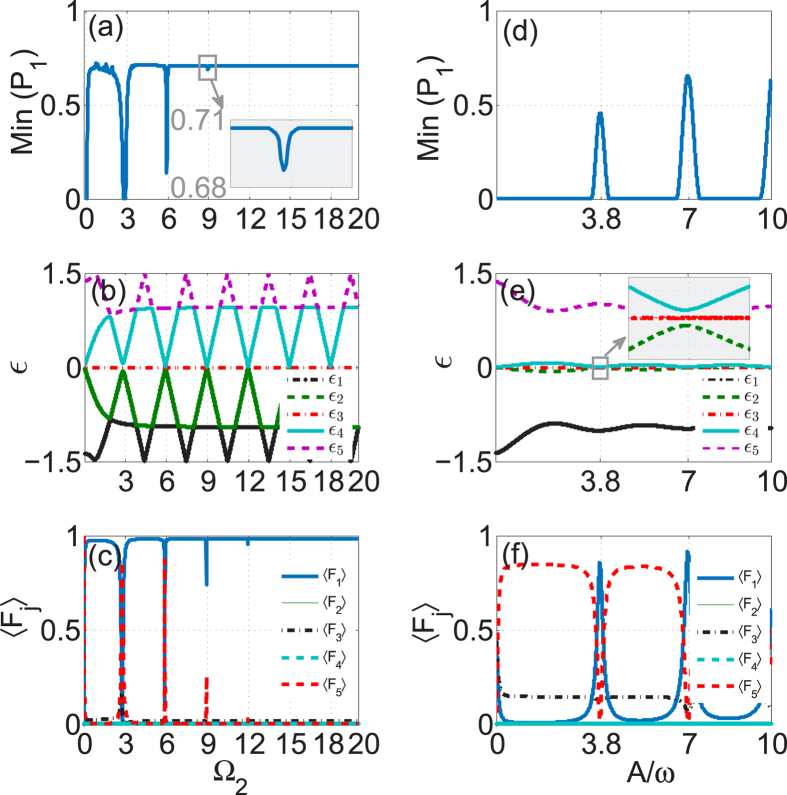
PAT in five-guide optical system. The left column: (**a**) the minimum value of population distribution at guide 1, Min(*P*_1_), versus Ω_2_ with *A* = 6.6, *ω* = 3, Ω_1_ = 1; (**b**) the corresponding quasi-energy *ε* versus Ω_2_ and (**c**)the time-averaged population 〈*F*_*j*_〉 belonging to the zero-energy (dark) Floquet state versus Ω_2_. The right column: (**d**) Min(*P*_1_) versus *A*/*ω* for the 1-photon resonance *ω* = 3, Ω_2_ = 2.8, Ω_1_ = 1; (**e**) the corresponding quasi-energy *ε* versus *A*/*ω* and (**f**) the time-averaged population 〈*F*_*j*_〉 belonging to the zero-energy (dark) Floquet state versus *A*/*ω*.

We now turn to the case of the five-guide optical system and investigate the beam dynamics by direct integration of the time-dependent Schrödinger equation (1) (*j* = 2, 3 and *N* = 5) with the light initially localized at the guide 1. In Fig. 5, fixing the parameters A = 6.6, *ω* = 3, Ω_1_ = 1 as before, we show the value of Min(*P*_1_) as a function of Ω_2_ which exhibits a sequence of PAT resonances with similar behavior as that of a three-guide system. The higher *n*-photon resonances with *n* ≥ 3 become very weak, almost invisible, as illustrated in the inset of Fig. 5(a). By comparison of Fig. 2(a) with Fig. 5(a), it is apparent that the same order PAT resonance for the five-guide system is much narrower and weaker than for the three-guide system. Like the case of the three-guide system, this five-guide system also possesses a dark Floquet state with zero quasi-energy and negligible population at all of the even *j*-th guides (modes), as illustrated in Fig. 5(b,c). Reason for the existence of the analog of PAT resonances in the five-guide system lies in that population distribution 〈*F*_1_〉 for the dark Floquet state simultaneously displays a series of sharp dips at the positions of PAT resonances [see Fig. 5(c)].

In Fig. 5(d), we plot Min(*P*_1_) obtained in the five-guide system as a function of the modulation parameter *A*/*ω* for the 1-photon resonance Ω_2_ = 2.8, *ω* = 3, Ω_2_ ≈ *ω*. As discussed before, we can observe that the values of Min(*P*_1_) are peaked at the zeros of *J*_1_(*A*/*ω*), at which CDT occurs, while between the peaks Min(*P*_1_) take extremely low values as result of PAT. However, compared with the case of three-guide system, the peaks in Min(*P*_1_) are considerably lower and broader. As can be clearly seen from Fig. 5(e,f), the peaks in Min(*P*_1_) are indeed centered at the points of closest approach of the quasi-energies where the dark Floquet state has a population 〈*F*_1_〉 > 0.5. The numerical results establish again a firm link between PAT and dark Floquet state in our considered systems.

### Tunneling dynamics in the four- and six-guide optical systems and beyond

**Figure 6:**
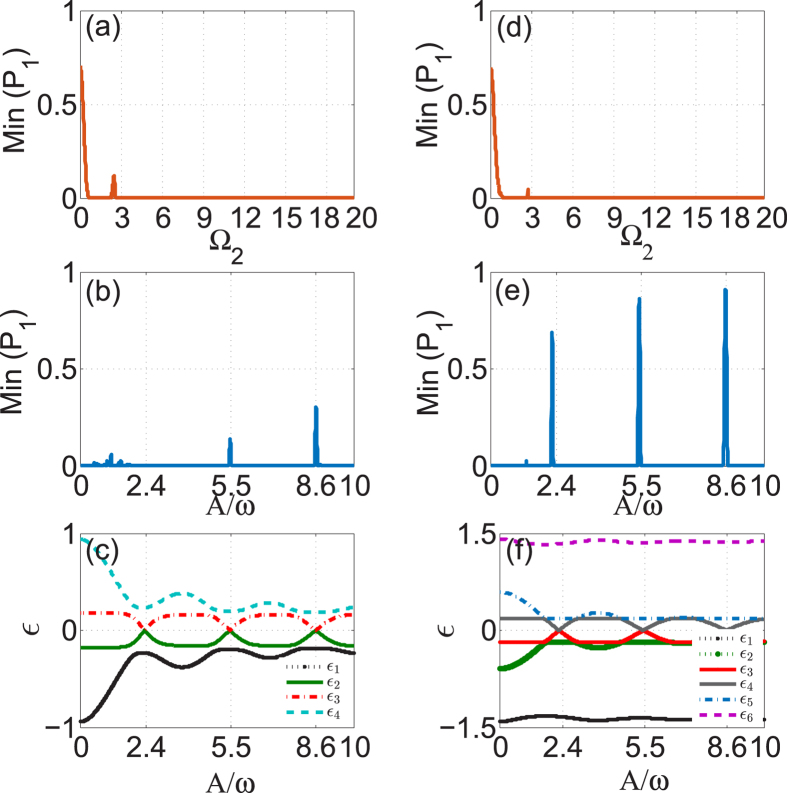
The left column: the characteristics of four-guide optical system; The right column: the characteristics of six-guide optical system. (**a**,**d**) Min(*P*_1_) versus Ω_2_ at *A* = 6.6, *ω* = 3, Ω_1_ = 1; (**b**,**e**): Min(*P*_1_) versus *A*/*ω* at Ω_2_ = 3, *ω* = 3, Ω_1_ = 1; (**c**,**f**): quasi-energies *ε* versus *A*/*ω* at Ω_2_ = 3, *ω* = 3, Ω_1_ = 1.

Finally, we briefly discuss the case of the four- and six-guide systems. The dynamics for *N* = 4 and *N* = 6 are presented in Fig. 6 on the basis of a full numerical analysis of Equation (1) with the light initially populated in the guide 1. It tells the existence of a sharp transition from CDT to complete tunneling for both cases of *N* = 4 and *N* = 6 when the coupling strength Ω_2_ is increased from zero. A close examination of the tunneling dynamics at Ω_2_ = n*ω* shows that the value of Min(*P*_1_) displays narrow peaks nearly at zeros of *J*_0_(*A*/*ω*) where a pair of quasi-energies become degenerate. This closely resembles the case of the high-frequency modulation *ω* ≫ max(Ω_1_, Ω_2_) where CDT is dominated by the zeros of *J*_0_(*A*/*ω*). As shown in Fig. 6(b,e), the localization centered nearly at zeros of *J*_0_(*A*/*ω*) is fairly smaller for the four-guide system, but still generates high peaks for the six-guide system.

Moreover, we have simulated multiwaveguide systems of other numbers of waveguides. The numerical results, which are not displayed here, show that the PAT resonances probably occur in all the odd-*N*-guide optical systems, while the PAT resonances become weaker with the increase of number of guides. However, all the even-*N*-guide optical systems exhibit CDT to complete tunneling transition without appearance of *n*-photon-like resonance when the coupling strength Ω_2_ is increased from zero, which is totally different from the case of odd-*N*-guide system.

### Possibility of experimental realization

Now, we discuss the experimental possibility of observing our theoretical predictions based on the coupled-mode equations. A more rigorous dynamics for our system can be simulated by the Schrödinger equation for the dimensionless field amplitude *E*, which describes the light propagation along the *z* axis of an array of *N* waveguides^25^,^26^


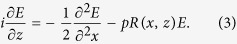


Here *x* and *z* are the normalized transverse and longitudinal coordinates, *p* describes the peak contrast (variation) in the refractive index between the unmodulated guiding structure and the substrate. The dimensionless variables *x*, *z* and *p* are related to the corresponding physical quantities *x*′, *z*′ and *p*′ by the scaling 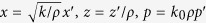, where 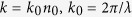, with *λ* being the vacuum wavelength of light and *n*_0_ the reference (substrate) index, and *ρ* represents a reference length which is chosen to be of the order of 10^−3^m in the experiments^25^,^26^. The normalized dimensionless power is defined by 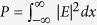. After a transformation of dimensionless field intensity to the physical field intensity defined by 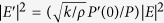, we get the physical input power (in W/m) 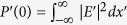. Therefore, the dimensionless field amplitude *E* is normalized in unit of 
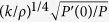
. For our system, the refractive index of the first waveguide is harmonically modulated along the propagation direction, while all other *N*−1 waveguides are unmodulated. The corresponding refractive index distribution of this kind of waveguide system is given by


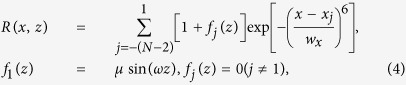


with the position of each waveguide being *x*_*j*_, the channel width *w*_*x*_, the longitudinal modulation amplitude *μ*, and the modulation frequency *ω*. Therein the super-Gaussian function 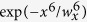 describes the profile of a single waveguide with width *w*_*x*_. In our discussion, all the waveguide spacings [*x*_*j*_ − *x*_*j*−1_] are identical except that the spacing between the bottom boundary waveguide and its neighbor is variable.

In what follows, we will illustrate our main results with a triplet waveguide system (*N* = 3) by directly integrating the field propagation Equation (3) with realistic experimental parameters. We set *w*_*x*_ = 0.3, *p* = 2.78, *μ* = 0.2 and *ω* = 3.45 × (*π*/100). We characterize two distinct waveguide spacings as *w*_1_ and *w*_2_ respectively, where *w*_1_ = *x*_1_ − *x*_0_ stands for the separation between the top waveguide and the middle waveguide and *w*_2_ = *x*_0_ − *x*_−1_ the separation between the bottom waveguide and the middle waveguide. Further we set *w*_1_ = 3.2 and choose different values of *w*_2_ to observe PAT resonance. As in the experiments^25^,^26^, *w*_*x*_ and *x*_*j*_ are in units of 10 *μ*m, and *p* = 2.78 corresponds to a real refractive index of 3.1 × 10^−4^ (weakly guided). The small change of index between the guide (core) and the substrate, which can be fabricated by using a femtosecond-writing method^27^, allows the weak-guidance approximation to be employed for obtaining the scalar wave equation (3). In all simulations we excited the top channel at *z* = 0, using the fundamental linear mode of the isolated waveguide. It is instructive to normalize the modulation frequency to the beating frequency of the unmodulated linear dual-core coupler with spacing *w*_1_, Ω_*b*_ = 2Ω_1_ = 2π/*Z*_*b*_, where *Z*_*b*_ is a beating period representing the shortest distance for the light returning to the input waveguide. For our set of parameters one has *Z*_*b*_ = 100 and thus *ω* = 3.45 Ω_1_.

**Figure 7:**
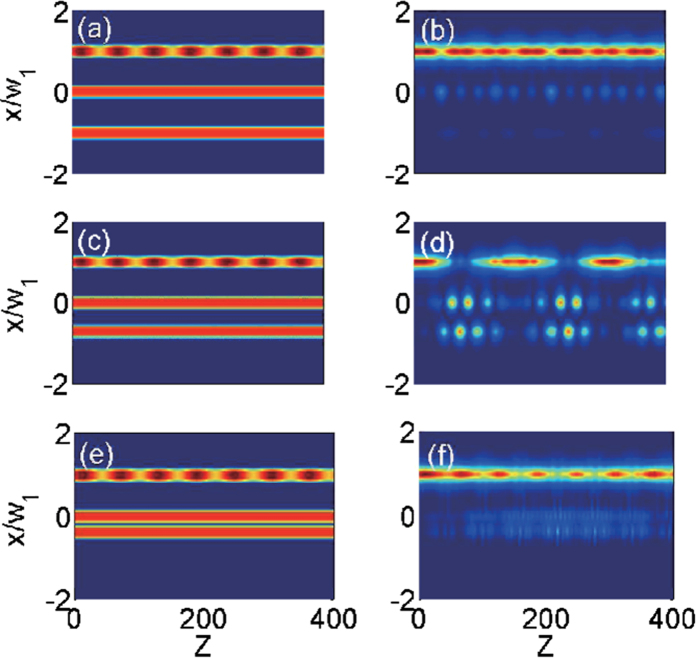
Light propagation in three-guide optical systems with different spacings between the bottom and the middle waveguide for the input beam centered at the top waveguide. First row: (**a**,**b**): the refractive index distribution *R*(*x*, *z*) and the light propagation |*E*(*x*, *z*)|^2^ for a three-guide system with equal channel spacing *w*_1_ = 3.2; Second row: (**c**,**d**): the refractive index distribution *R*(*x*, *z*) and the light propagation |*E*(*x*, *z*)|^2^ for a three-guide system with unequal channel spacing *w*_1_ = 3.2, *w*_2_ = 2.22; Third row: (**e**,**f**): the refractive index distribution *R*(*x*, *z*) and the light propagation |*E*(*x*, *z*)|^2^ for a three-guide system with unequal channel spacing *w*_1_ = 3.2, *w*_2_ = 1.2.

The beam dynamics of a three-guide optical system are visualized in Fig. 7 for three values of *w*_2_, which firmly verifies the predictions from the coupled-mode Equation (2). In Fig. 7. the left column shows the refractive index distribution *R*(*x*, *z*) and the right column shows the evolution of light intensity |*E*(*x*, *z*)|^2^ along the propagation direction. It can be readily observed from Fig. 7(a,b) that the light tunneling is almost completely suppressed, as the three-channel waveguide system has equal channel spacing *w*_1_ = *w*_2_ = 3.2. At *w*_2_ = 2.22, the light coupling between the waveguide channels is restored [see Fig. 7(c,d)]. The revival of light tunneling is a signature of PAT resonance predicted by the coupled-mode theory. In fact, our numerical simulation (not shown here) reveals that the beating period of an unmodulated linear dual-core coupler with a channel spacing 2.22 is about 100/3.45. As such, we have Ω_2_ ≈ 3.45Ω_1_ and Ω_2_ ≈ *ω*, which is in fact the position of the first photon resonance. As the channel spacing *w*_2_ is reduced further, it is expectable to observe again the strong suppression of light tunneling [see Fig. 7(e,f)]. These results are in good qualitative agreement with those in Fig. 2(a) based on the coupled-mode equation.

## Discussion

We have theoretically reported an analog of PAT in a three-channel waveguide system, in which the space separation between the bottom and the middle waveguides is adjustable and the refractive index of the top waveguide is modulated periodically along the light propagation direction. With the standard coupled-mode theory, the system can be described by a driven three-state discrete model with two distinct coupling strengths Ω_1_ and Ω_2_, where Ω_1_ stands for the coupling strength between states 1 and 2, and Ω_2_ between states 2 and 3. In studying the three-state discrete model, we have found that (i) a strong suppression (CDT) associated with the zero-energy (dark) Floquet state persists even in the non-high-frequency modulation regimes where *ω* ≤ max(Ω_1_, Ω_2_) except at a series of resonance positions; (ii) at particular values of the coupling strength Ω_2_, Ω_2_ ≈ *nω* with *n* being integer, the tunneling dynamics is (partly) restored, analogous to the *n*-photon-like resonances which overcome the effect of suppression of tunneling. The numerical calculations illustrate that the PAT resonances exist in the five-state system and also probably exist in the systems with arbitrary odd number of coupled states. In particular, the PAT resonances will become weaker with the increase of number of states (modes). This type of PAT found in our work has a different origin from traditional PAT. It is closely related to the existence of the dark Floquet state. The main results are demonstrated by the direct numerical simulations of propagation dynamics based on the full continuous model with realistic experimental parameters, which indicates that the PAT found in our work can be readily tested in the current experimental setup. Because of the equivalence between the Schrödinger equation and the optical wave equation, we select the engineered photonic lattice as our model system due to its robust and feasibility in the realistic experiment and, obviously, our results can also be applied to the quantum systems such as cold atom or trapped ion in optical lattices and electron transport in quantum dot chains.

## Methods

To calculate the Floquet states and corresponding quasienergies of the coupled-mode Equation (1), we carry out a procedure that runs in principle as follows. We first numerically integrate Eq. (1) from *z* = 0 to *T* with the initial conditions *a*_*j*_(0) = *δ*_*jm*_, *m* = 1, 2, 3, … *N*. The time evolution operator over one modulation period *U*(*T*, 0) is given by a *N* × *N* propagator matrix, where the matrix element *U*_*j,m*_ is the value *a*_*j*_(*T*) obtained by solving Eq. (1) with the initial condition *a*_*l*_(0) = *δ*_*jm*_. Given that the Floquet states are eigenstates of *U*(*T*, 0) with eigenvalues exp(−*iεT*), the quasienergies and corresponding Floquet eigenmodes are numerically computed by direct diagonalization of *U*(*T*, 0).

In our numerical simulations, the coupled-mode equation is numerically solved with Runge-Kutta method and the simulations of propagation dynamics based on the continuous wave equation are implemented with the use of the split-step Fourier method. In the meantime, imaginary-time evolution method is used in finding the fundamental mode of the isolated top waveguide which is taken as input beam for numerical demonstrations of PAT in the continuous model.

## Additional Information

**How to cite this article**: Li, L. *et al.* An analog of photon-assisted tunneling in a periodically modulated waveguide array. *Sci. Rep.* **6**, 35744; doi: 10.1038/srep35744 (2016).
